# Modification of the Langendorff system of the isolated beating heart for experimental radiotherapy at a synchrotron: 4000 Gy in a heart beat

**DOI:** 10.1107/S1600577522004489

**Published:** 2022-05-18

**Authors:** Elisabeth Schültke, Michael Lerch, Timo Kirschstein, Falko Lange, Katrin Porath, Stefan Fiedler, Jeremy Davis, Jason Paino, Elette Engels, Micah Barnes, Mitzi Klein, Christopher Hall, Daniel Häusermann, Guido Hildebrandt

**Affiliations:** a Department of Radiooncology, Rostock University Medical Center, Südring 75, 18059 Rostock, Germany; b Centre for Medical Radiation Physics, University of Wollongong, Wollongong, Australia; c Oscar Langendorff Institute of Physiology, University of Rostock Medical Center, Rostock, Germany; d Center for Transdisciplinary Neurosciences Rostock, Rostock, Germany; e European Molecular Biology Laboratory, Notkestrasse 85, 22607 Hamburg, Germany; f Australian Synchrotron/ANSTO, Clayton, Australia

**Keywords:** microbeam irradiation (MBI), Langendorff model of the isolated beating heart, organ of risk

## Abstract

Microbeam radiotherapy could help to cure malignant tumours which are currently still considered therapy-resistant. With an irradiation target in the thoracic cavity, the heart would be one of the most important organs at risk. To assess the acute adverse effects of microbeam irradiation in the heart, a powerful *ex vivo* tool was created by combining the Langendorff model of the isolated beating mammalian heart with X-Tream dosimetry. In a first pilot experiment conducted at the Biomedical and Imaging Beamline of the Australian Synchrotron, the system was tested at a microbeam peak dose approximately ten times higher than the anticipated future microbeam irradiation treatment doses. The entire heart was irradiated with a dose of 4000 Gy at a dose rate of >6000 Gy s^−1^, using an array of 50 µm-wide microbeams spaced at a centre-to-centre distance of 400 µm. Although temporary arrhythmias were seen, they reverted spontaneously to a stable rhythm and no cardiac arrest occurred. This amazing preservation of cardiac function is promising for future therapeutic approaches.

## Abstract 2

The modification of the Langendorff model of the isolated beating heart for microbeam irradiation studies and the results of the first pilot experiment, conducted at the Biomedical and Imaging beamline (IMBL) of the Australian Synchrotron, are reported.

## 1. Introduction

Microbeam irradiation (MBI) has shown much promise in pre-clinical studies to significantly improve tumour control in cases where current clinical radiotherapy approaches fail (Potez *et al.*, 2020 ▸; Schültke *et al.*, 2017 ▸; Bouchet *et al.*, 2016 ▸; Laissue *et al.*, 2007 ▸). Two hallmarks, which distinguish MBI from the clinically already established radiotherapy approaches, are an ultrafast dose deposition and the spatial dose fractionation at the micrometre range. Typical MBI dose rates are ≥100 Gy s^−1^, compared with clinical dose rates of 6–20 Gy min^−1^. A multislit collimator (MSC) is used to generate an irradiation field of quasi-parallel microbeams, covering the target zone with a grid of alternating high dose (peak) zones and low dose (valley) zones. Peak doses are typically several hundred Gy, administered in one single fraction. Thus, the peak doses are higher by one or even two orders of magnitude than single fraction doses in clinical radiotherapy. MBI used with therapeutic intent has become known as microbeam radiotherapy (MRT).

In the early years of MRT research, the main focus was almost exclusively on the brain. More recently, however, lung carcinomas have also been considered potential targets (Schültke *et al.*, 2021 ▸; Trappetti *et al.*, 2021 ▸). In this case, the heart would be one of the most important organs at risk. Therefore, we have designed an *ex vivo* model system to investigate the effects of high MBI peak doses on the electric impulse conducting system of the heart. Furthermore, we tested the system at an MBI peak dose which is approximately ten times higher than the peak doses anticipated in future MRT studies.

## 2. Material and methods

### 2.1. Experimental setup

In this study, one major challenge was the lateral movement of the beating heart because this physiologic movement of the irradiation target might lead to smearing of the microbeam edges. A distinct separation of peak and valley dose zones is required for a high peak-to-valley dose ratio (PVDR). Smearing of the beam edges due to physiologic movement would lead to a decrease of the PVDR and subsequently may result in an impairment of normal tissue function.

The risk of beam smearing is reduced proportionally to the irradiation time. Our aim was to perform the entire irradiation within the period of roughly one heart beat. Therefore, the MBI dose rate should be as high as possible to keep the proportion of a cardiac cycle used for irradiation as low as possible.

A fixed-space multislit collimator (UNT, Morbier, France) was inserted into the incident beam, generating an array of quasi-parallel microbeams of 50 µm width spaced at a centre-to-centre distance of 400 µm. In the tissue, this generated a repetitive sequence of peak dose (high dose) and valley dose (low dose) zones.

EBT3 Gafchromic film (Ashland, Bridgewater, USA) was used to verify the uniformity of the microbeams and the correct positioning of the heart with respect to the irradiation field.

#### 2.1.1. Dose simulation, dosimetry and determination of the optimal beam filtration

Contrary to clinical radiotherapy with a linear accelerator (LINAC), where the treatment beam is moved around the irradiation target, at the synchrotron the beam is in a fixed position. When the irradiation target (treatment field) in its *z*-axis is larger than the static beam, the target is moved vertically through the beam during dose deposition. Dose delivery is controlled by varying the speed of the vertical translation through the beam. Considering the maximum possible vertical translation speed of 20 mm s^−1^ and a wiggler strength of 4 T, we compared the two beam filtration systems available at the synchrotron beamline: (1) combined paddles of 2.82 mm effective thickness aluminium and 1.41 mm effective thickness copper (CuAl filtration), and (2) two aluminium paddles each of 2.82 mm effective thickness (AlAl filtration) (Stevenson *et al.*, 2017 ▸).

A beam of 30 mm × 1.053 mm (horizontal × vertical) has been shaped by a beam-defining aperture (BDA). Parasitic scattering was removed by a series of collimating slits. A conformal mask of dimensions of 20 mm × 20 mm (Fig. S1 of the supporting information) limited the actual irradiation field when an object was moved vertically through the beam.

Two complementing detectors were used to characterize the treatment field: a small-volume PTW model 31014 ionization chamber (IC) and a single strip detector (SSD) (Davis *et al.*, 2018 ▸). The IC is routinely used on the Imaging and Biomedical Beamline (IMBL) of the Australian Synchrotron and is calibrated in kilovoltage beams at PTB, Germany, although is not suitable for microbeam dosimetry. The SSD offers a spatial resolution of approximately 10–15 µm (Lerch *et al.*, 2011 ▸). When combined with the X-Tream dosimetry system, it also offers real-time signal read-out, and is the only system on the market which allows the monitoring of dose deposition during the irradiation process in a single microbeam (Petasecca *et al.*, 2012 ▸; Fournier *et al.*, 2016 ▸; Davis *et al.*, 2019 ▸). However, the SSD has no calibration traceable to a primary standard.

Cross-calibration of the IC and SSD detectors was performed for each filtration modality at the centre of a 25 mm × 25 mm × 55 mm RMI457 Gammex Solid Water phantom with both detectors exposed to identical 20 mm × 20 mm uniform fields (the smallest size that is recommended for the IC). These measurements were confirmed against a GEANT4 Monte Carlo simulation (Dipuglia *et al.*, 2019 ▸; Engels *et al.*, 2020 ▸) of the same phantom geometry and irradiation conditions.

**Figure 1:**
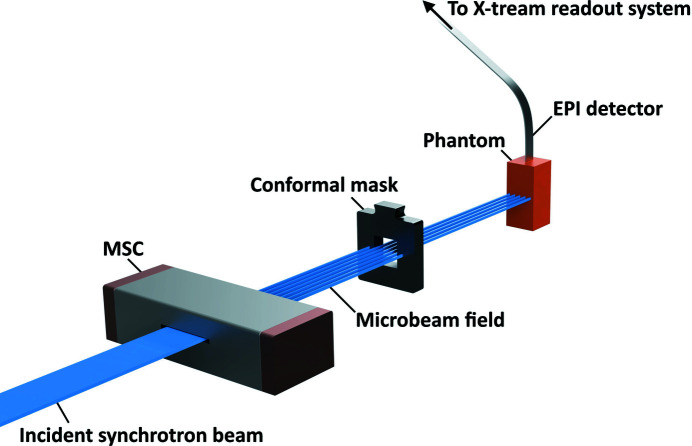
Experimental setup for X-Tream dosimetry as part of the procedure used to calibrate simulations to experimental dose rates measured with the X-Tream system.

Following calibration and insertion of the multislit collimator (MSC) to generate the microbeam array, an intrinsic and Step-and-Scan (SnS) measurement was performed with the SSD at 12.5 mm depth within the 25 mm × 25 mm × 55 mm solid water phantom. The full microbeam field was captured with each peak and valley region captured in detail. The setup for X-Tream dosimetry is depicted schematically in Fig. 1 ▸.

The heart model measured 12 mm × 12 mm × 18 mm, slightly smaller than the 25 mm × 25 mm × 55 mm phantom. To account for the difference in model size versus phantom size, the now calibrated GEANT4 simulation was used to simulate a uniform solid water phantom with dimensions matching the measurements of the heart. The simulated MBI peak dose rate at the surface of the phantom (here defined as the average dose to a 0.5 mm voxel centred at 0.25 mm depth) was then used to calculate the translation velocity required to achieve the desired dose of 4000 Gy.

### 2.2. Modification of the Langendorff model of the isolated beating heart

The functional principle of the Langendorff system, developed by Oscar Langendorff at the Institute of Physiology of the Rostock University in Germany in 1895 (Bell *et al.*, 2011 ▸), is based on a retrograde perfusion of the heart with an oxygenated temperature-regulated fluid via the aorta. The retrograde fluid pressure forces the aortic valve shut and the coronary arteries are perfused with the oxygenated fluid. We have modified the Langendorff system (ADInstruments Ltd, Bella Vista, Australia) for use at the IMBL of the Australian Synchrotron. The experiments were conducted under the animal ethics permit AS2019_001.

The intrinsic *in vivo* heart rate of rats is 200–300 beats min^−1^. In order to simulate the conditions encountered in a human patient, the Langendorff system carrying hearts explanted after decapitation under deep anaesthesia from adult female Wistar rats was modified to decrease the heart rate to match that of a resting human patient.

Under temperature-controlled conditions (32°C), three primary functional parameters of the heart were recorded employing the *LabChart 7.3.7* software pack (ADInstruments Ltd, Bella Vista, Australia) at a sampling rate of 1 kHz. The coronary perfusion pressure was determined using a pressure transducer (ADInstruments) close to the cannula inserted in the aorta. A fluid-filled latex balloon (ADInstruments) connected to a pressure transducer was placed in the left ventricle to measure the left ventricular pressure. A bipolar electrocardiogram (ECG) was recorded with two electrodes inserted into the right and left ventricle for approximately 35 min prior to irradiation to obtain baseline values. After irradiation, ECG and ventricular pressure were recorded for another 60–90 min.

Following dose simulation and measurements by X-Tream dosimetry, we modified the Langendorff heart preparation to record representative functional parameters of the cardiac impulse conduction system like the coronary resistance and the left ventricular pressure before, during and after MBI.

In order to test the cardiac functional reserves, the hearts were challenged twice with norepinephrine (NE, 10^−5^ mol l^−1^; Sigma-Aldrich, Taufkirchen, Germany), once before and once approximately 50–60 min after irradiation.

### 2.3. MBI of the isolated beating heart

Two hearts were irradiated completely with an array of quasi-parallel microbeams with an individual beam width of 50 µm, spaced at a centre-to-centre distance of 400 µm and with an MBI peak dose of 4000 Gy. This dose was at least ten-fold higher than the expected peak dose in future preclinical studies. To administer this MBI peak dose of 4000 Gy, the vertical translation speed was 3.45 mm s^−1^. Two additional hearts served as non-irradiated controls.

**Figure 2:**
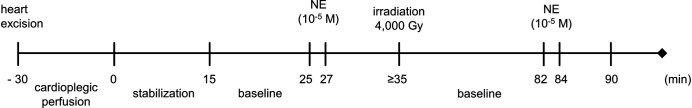
Experimental protocol of the heart preparation in the Langendorff setup at the IMBL beamline. After a short cardioplegic phase, the hearts were supplied with oxygenated perfusion solution. The hearts were stimulated with NE (10^−5^ *M*) once before and once after the irradiation to determine the cardiac reserve.

After the end of the irradiation experiments, the hearts were fixed, mounted, sectioned and immunostained with anti-gamma H2AX antibody (Abcam, ab2893, Cambridge, UK) to visualize the DNA double-strand breaks (Fernandez-Palomo *et al.*, 2015 ▸). The experimental paradigm is depicted in Fig. 2 ▸.

## 3. Results

### 3.1. Dosimetry and beam filtration

With aluminium-only filtration, the dose rate was 6898.6 Gy s^−1^ and thus approximately six times higher than the dose rate obtained with copper–aluminium filtration (1032.4 Gy s^−1^). The dose rates measured with the X-Tream system were sufficiently close to the simulated values.

Thus, the AlAl filtration mode proved to be best suited to obtain the goal of our study: to administer a 4000 Gy peak dose within one single cardiac cycle (heart beat), assuming a pulse frequency of approximately 60 beats min^−1^ or less, which would correspond to the resting heart rate of a human patient. The irradiation field measured 20 mm × 20 mm and contained 50 microbeams. The valley dose varied through the depth of the heart. Based on the GEANT4 simulations, the heart received a minimum valley dose of 50 Gy and a maximum valley dose of 75 Gy. Thus, the resulting PVDR was between 1:80 and 1:53.

### 3.2. MBI of the Langendorff hearts

**Figure 3:**
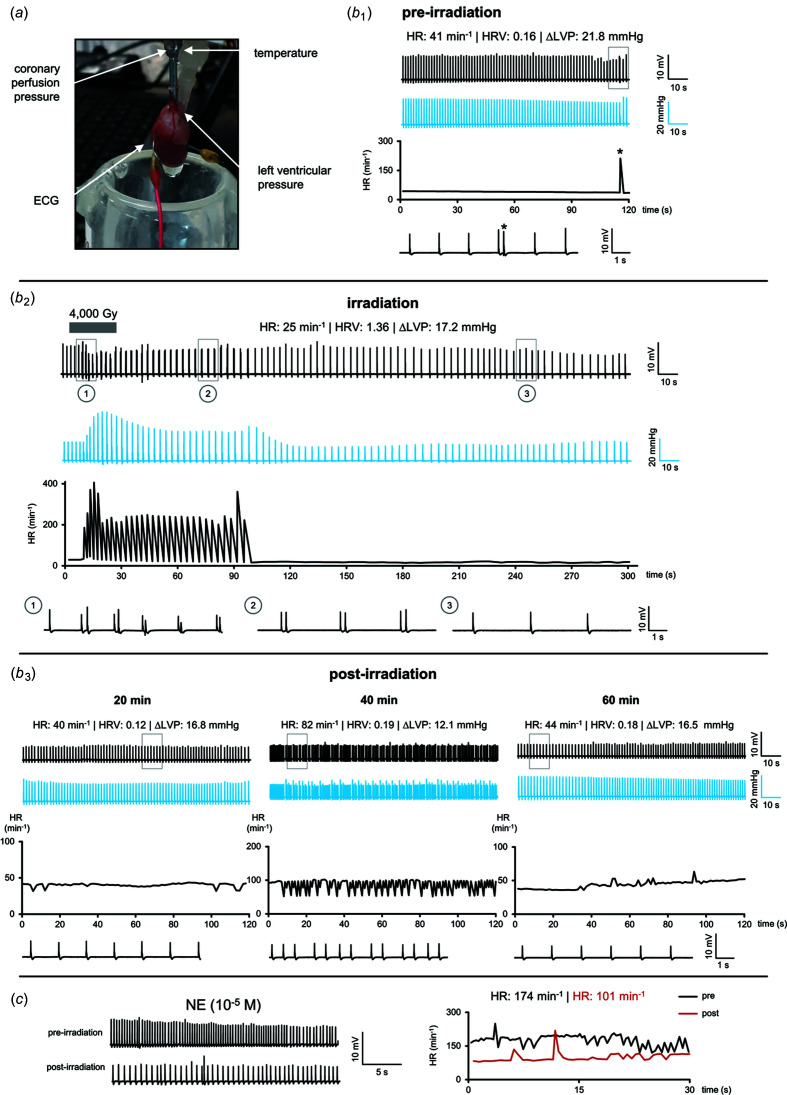
(*a*) Setup of the Langendorff heart, recording coronary perfusion pressure, temperature, electrocardiogram (ECG) and left ventricular pressure. (*b*_1_) ECG (black traces), left ventricular pressure (blue traces), time course of the heart rate (HR) and enlarged ECG during baseline before irradiation (HR; HRV: heart rate variability; ΔLVP: change in left ventricular pressure). (*b*_2_) ECG (black traces), left ventricular pressure (blue traces), time course of the heart rate (HR) and enlarged ECG during irradiation and early post-irradiation. A transient increase of left ventricular pressure was observed in this phase. (*b*_3_) ECG (black traces), left ventricular pressure (blue traces), time course of the HR and enlarged ECG at different post-irradiation time points. The heart rate completely returned to pre-irradiation baseline values within 60 min after MBI. (*c*) NE challenge ECG before and after irradiation (left) and heart rate (right). Norepinephrine (NE) still increased the heart rate after irradiation.

First, a stable baseline heart rate of 41 beats min^−1^ with a low heart rate variability (HRV; 0.16) was achieved [Fig. 3 ▸(*b*_1_)]. Occasionally, ventricular extrasystoles were observed, most likely due to the low temperature of 32°C [see asterisks in the time course and in the enlarged inset, Fig. 3 ▸(*b*_2_)].

After complete irradiation of the heart with an array of microbeams at peak doses of 4000 Gy, no cardiac arrest occurred. Temporary arrhythmias were seen during and within 30 min after irradiation. However, severe arrhythmias, such as ventricular tachycardia or fibrillations, were not observed.

Within the 90 min follow-up period, an almost complete recovery to a stable heart rate occurred. The electrophysiological maximum voltage amplitudes, known as QRS complexes, remained reduced after irradiation, indicating that parts of the ventricular myocardium were silenced. Cardiac output as assessed by the left ventricular pressures was reduced from 22 to 17 mmHg [Fig. 3 ▸(*b*_3_)].

We repeated this experiment with a second Langendorff heart preparation. In this specimen, we found a heart rate of 75–85 beats min^−1^ before irradiation with an MBI peak dose of 4000 Gy, which dropped to 72 beats min^−1^ during irradiation, but without showing any arrhythmia instantly. During the early follow-up, within 30 min after irradiation, we again observed the tendency towards lower heart rates (between 57 and 75 beats min^−1^), but also an increased incidence of ventricular extrasystoles with partly occurring bigeminy rhythms. Again, the QRS morphology did not change, indicating that the physiological intraventricular conduction system was preserved. At 60 min follow-up, we found complete recovery with a stable heart rate of 100–120 beats min^−1^.

In our *ex vivo* experiment, irradiation immediately led to arrhythmia, in this case bigeminy, which is defined as regular contractions followed by a premature ventricular contraction [see Fig. 3 ▸(*b*_2_), plot 1]. An increase in the heart rate was observed. After approximately 2 min, the ventricular extrasystoles stopped and the heart fell back into a pacemaker-driven rhythm. The spontaneous disappearance of the arrhythmia suggests that it were caused by temporary dis­arrangements at the level of plasma membrane and membrane-bound proteins (*e.g.* ion channels and ion pumps which maintain the ion flow across the membrane) rather than by more permanent structural damage to the cardiac muscle itself.

Both of the two non-irradiated hearts showed rather stable baseline heart rates and responded well to the NE-challenge (asterisks in Fig. S1 of the supporting information). An increasing incidence of ventricular extrasystoles was observed over time, in both irradiated and non-irradiated hearts, indicating that they were not caused by MBI.

The NE-challenge in the two non-irradiated control hearts resulted in similar accelerations before (168–175 beats min^−1^) and after sham-irradiation (173–196 beats min^−1^). In the irradiated hearts, for comparison, the maximum response after irradiation was reduced by approximately 35%. Only minor changes in the coronary resistance were observed during irradiation (increase of 2.5%) and after irradiation (increase of 12.5%).

**Figure 4:**
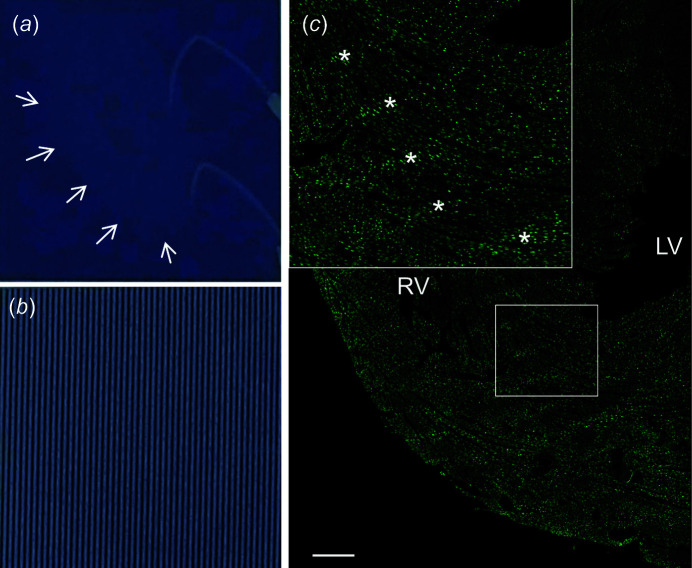
Heart contour (white arrows) and ECG electrodes (*a*) and complete microbeam array (*b*) registered on radiosensitive Gafchromic film [the films are of the same size in (*a*) and (*b*)]. (*c*) Gamma H2AX immunostain showing the DNA double-strand breaks in the heart tissue. The bright green fluorescent dots represent nuclei harbouring the phospho­rylated histone H2A in response to DNA double-strand breaks. RV: right ventricle; LV: left ventricle. Scale bar = 500 µm. Asterisks in the enlargement indicate traces of DNA double-strand breaks in the microbeam paths. The γH2AX-positive nuclei between the microbeam paths could be partially due to differences between the patterns of muscle contraction at the time of irradiation and the time of sample fixation and partially contributed by the valley dose.

In the heart muscle sections immunostained for γH2AX, the traces of the microbeams were visible as lines of DNA double-strand breaks, mirroring the microbeam geometry registered on radiosensitive Gafchromic film (Fig. 4 ▸).

## 4. Discussion

This was the internationally first study exploring the impact of extremely high MBI peak doses on electrical impulse conduction in the heart. With an MBI peak dose of 4000 Gy, we have chosen a dose approximately ten times higher than the maximum dose to which the heart would be exposed in future *in vivo* studies and in clinical studies, respectively. Because this dose was higher by approximately two orders of magnitude, compared with clinical radiotherapy, it was difficult to predict acute radiogenic effects, including the risk of sudden cardiac death. Therefore, it appeared appropriate to obtain first data *ex vivo* but nevertheless in a functioning organ system.

The endpoint of this experiment was irreversible cardiac arrest. This endpoint was not reached. MBI at such a high peak dose may, however, affects the heart automaticity causing a brief temporary atrioventricular block. Although there was a decrease in amplitude, no changes in the morphology of the QRS complex, a typical early sign of functional deterioration, were observed. This indicates that, beyond the occurrence of self-limiting arrhythmias, the physiological normal intraventricular impulse conduction was preserved. More relevant signs of acute radiogenic toxicity were seen only when testing the functional reserve of the irradiated hearts.

The surprisingly low vulnerability of the cardiac impulse conduction system in this setup might be partially due to a FLASH effect, which describes the fact that dose deposition at ultra-high dose rates (>40 Gy s^−1^) results in surprisingly low normal tissue damage (Favaudon *et al.*, 2014 ▸, 2015 ▸).

In our study, the entire heart was exposed to MBI, which can be considered a worst-case scenario. In most clinical situations, the heart would be only partially exposed and receive only a fraction of the target treatment dose. Also, the maximum target dose would be ten times lower and therefore easy to administer within one single heart beat. Thus, the potential risk of MBI to impact on cardiac function would be significantly smaller than in our study.

While the data obtained in this study might not be statistically relevant, they suggest that we would not look at an extremely narrow therapeutic window when using MRT peak target doses up to 400 Gy in the thoracic cavity. It has already been shown that no significant lung fibrosis occurred with MRT peak doses of 400 Gy (Trappetti *et al.*, 2021 ▸). In this study, the heart was excluded from the irradiation field because no data were available about the response of the heart to MRT. Based on those observations, we now find it justifiable to propose an *in vivo* study with microbeam peak doses of up to 400 Gy to targets in the thoracic cavity with (most likely only partial) inclusion of the heart. Dose deposition in the heart is a relevant clinical scenario occurring when a central lung carcinoma is irradiated.

In summary, the combination of the Langendorff perfusion system of the isolated beating mammalian heart with X-Tream dosimetry is a powerful *ex vivo* tool to replace *in vivo* toxicity and dose-finding studies in the acute phase after experimental radiotherapy. Only once the range of safe treatment parameters has been identified are *in vivo* studies required for verification.

## Supplementary Material

Figure S1: heart rates over the entire observation period. DOI: 10.1107/S1600577522004489/tv5036sup1.pdf
