# Supplementary material for: Modification of the Langendorff system of the isolated beating heart for experimental radiotherapy at a synchrotron: 4000 Gy in a heart beat
Source: J Synchrotron Radiat. 2022 May 18;29(Pt 4):1027–32. doi: 10.1107/S1600577522004489 (PMC9255585; doi:10.1107/S1600577522004489)
Supplement: Supplementary file 1 [file s-29-01027-sup1.pdf]

JOURNAL OF SYNCHROTRON RADIATION

Volume 29 (2022)

Supporting information for article:

Modification of the Langendorff system of the isolated beating heart for experimental radiotherapy at a synchrotron: 4000 Gy in a heart beat

Elisabeth Schültke, Michael Lerch, Timo Kirschstein, Falko Lange, Katrin Porath, Stefan Fiedler, Jeremy Davis, Jason Paino, Elette Engels, Micah Barnes, Mitzi Klein, Christopher Hall, Daniel Häusermann and Guido Hildebrandt

4000 Gy

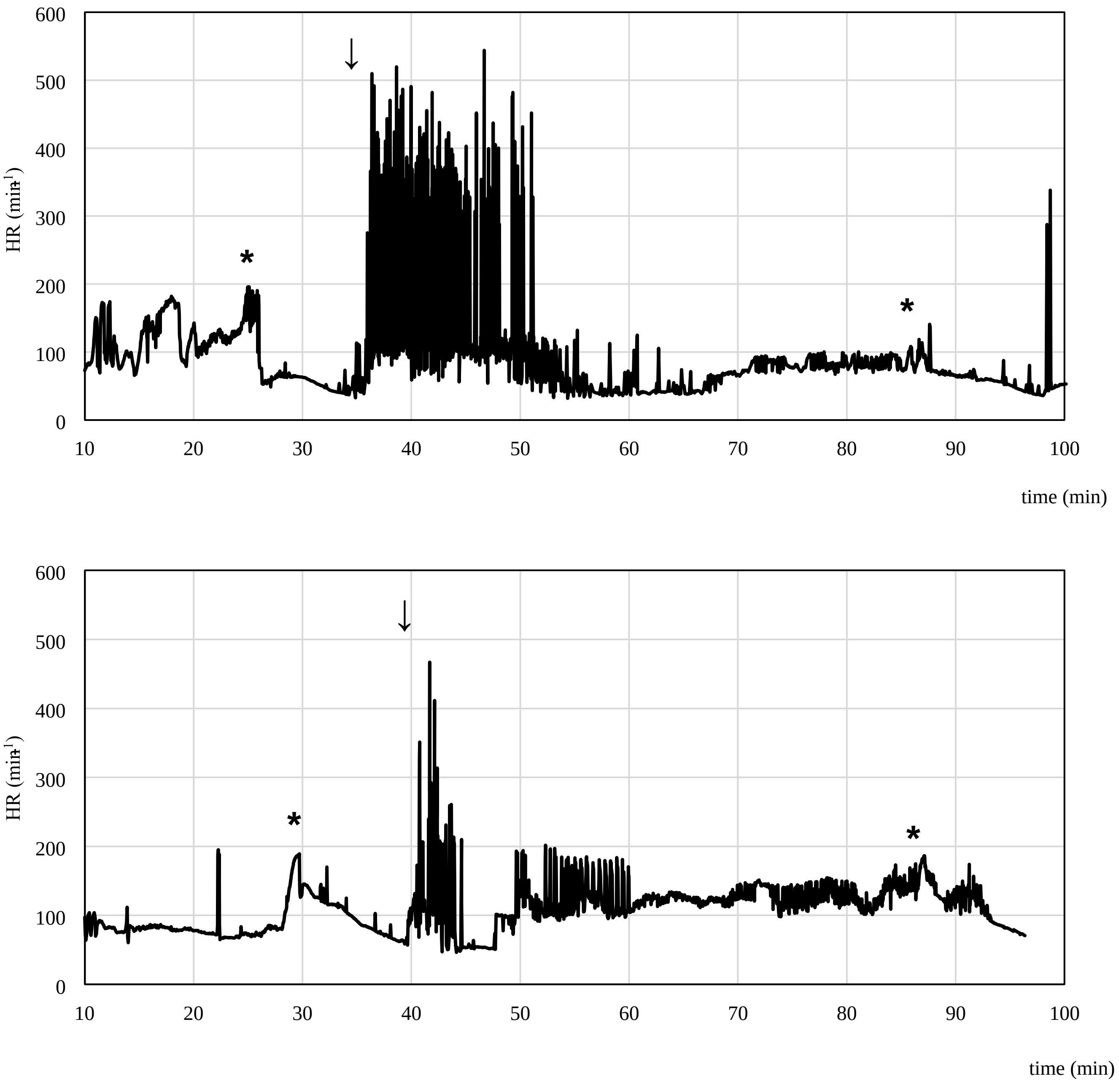

↓ : irradiation ; * : NE stimuation

non-irradiated controls

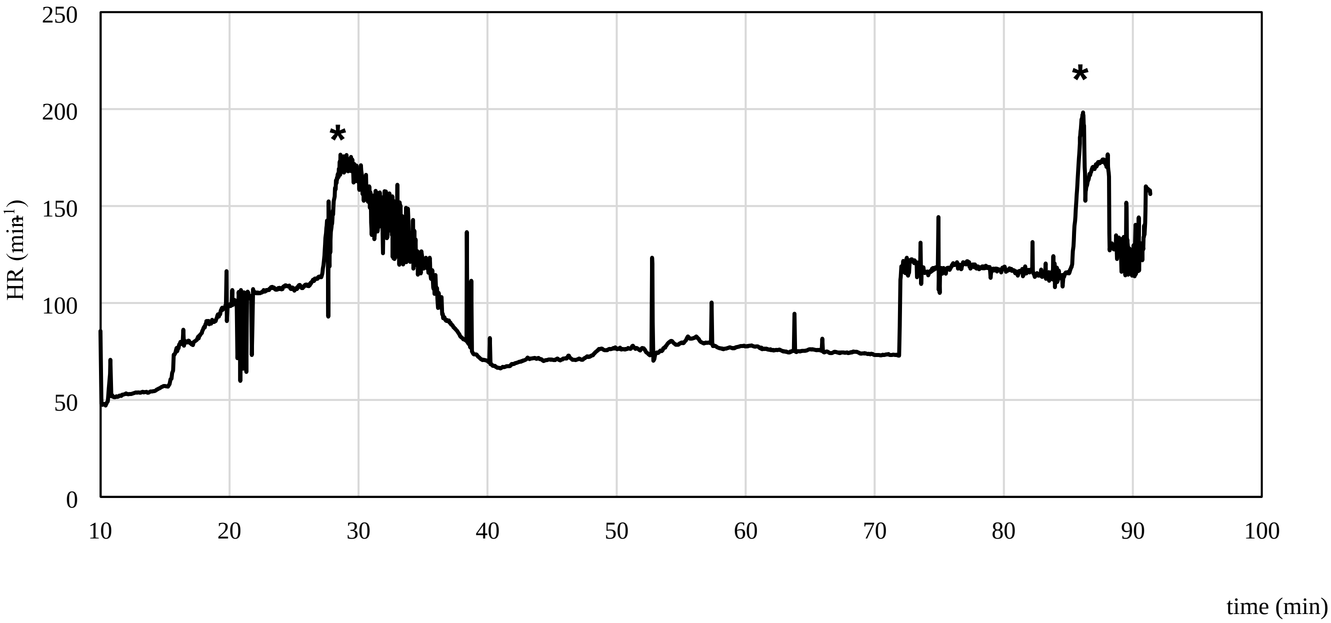

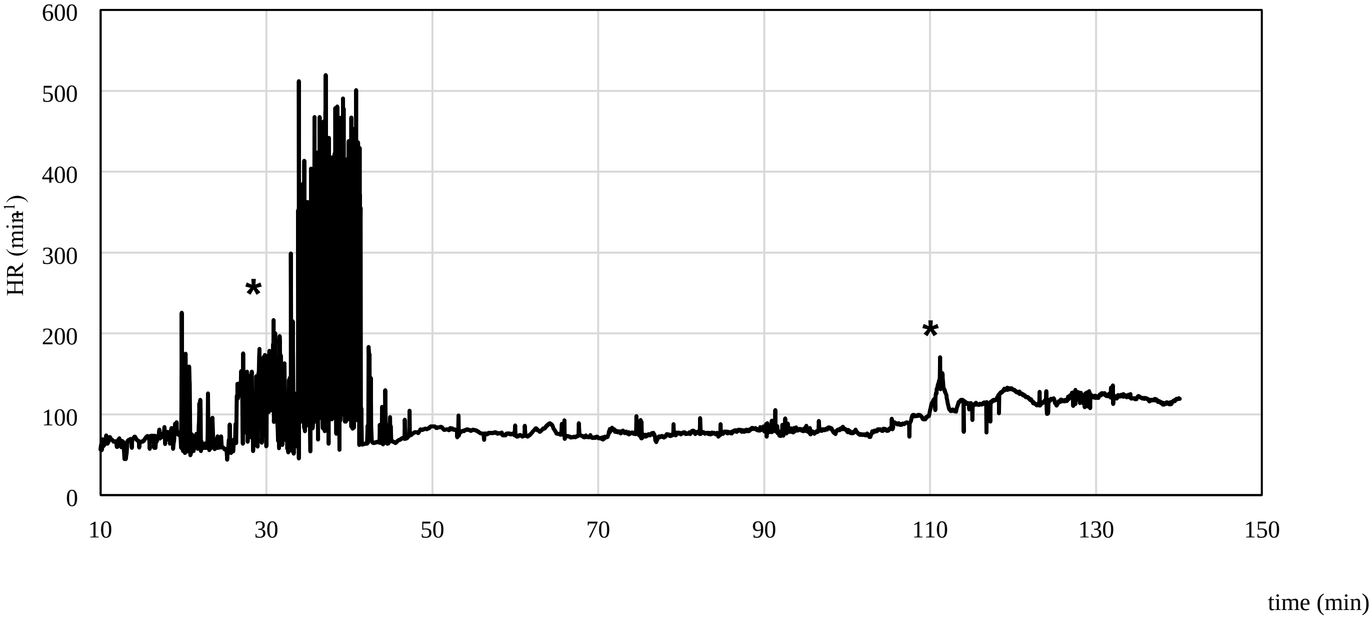

Figure S1 The heart rates over the entire observation period are shown for both hearts irradiated with 4000 Gy and both non-irradiated hearts. The arrow indicates the time of irradiation in the former two, the asterisk indicates the NE challenge in all preparations.
